# Less invasive surgery of the proximal aorta

**DOI:** 10.1186/1749-8090-8-S1-O36

**Published:** 2013-09-11

**Authors:** P Risteski, N Monsefi, T Josic, E Srndic, P Ilioska, A Moritz, A Zierer

**Affiliations:** 1 Department of Thoracic and Cardiovascular Surgery, Johann Wolfgang Goethe University, Frankfurt am Main, Germany

## Background

Partial upper sternotomy (PUS) is established less invasive approach for single and double valve surgery. Reports of aortic surgery performed through PUS are rare.

## Methods

The records of 52 patients undergoing primary elective surgery on the proximal aorta through PUS between 2005 and 2011 were reviewed. Patients mean age was 57 years, 35% were in NYHA Class III or IV, 59% had recent cardiac decompensation, and 17% had pulmonary hypertension. The PUS was taken down to the 4th left intercostal space in 44 patients (85%).

## Results

No conversion to full sternotomy was necessary. The aortic cross-clamp, cardiopulmonary bypass and operative times averaged 136 ± 20 min., 186 ± 36 min. and 327 ± 83 min., respectively. In eight patients, the right axillary artery was cannulated for establishing cardiopulmonary bypass; the others were cannulated centrally. All patients except one received a procedure on the ascending aorta, either replacement in 30 (58%) or reduction aortoplasty in 21 (40%). Aortic root replacement was additionally performed in 31 patients (60%), including David in 20 (38%) and Ross procedure in 6 (11.5%). The aortic arch was replaced either partially in 5 (10%) or totally in 3 (6%) patients, in moderate hypothermia employing antegrade cerebral perfusion. Additional procedures, included mitral valve repair in 15 (29%) patients and coronary grafting. Ventilation time, intensive care unit and hospital stay averaged 17 ± 12 hours, 2 ± 1, and 11 ± 9 days. Chest drainage was 470 ± 380 ml/24 hours. Permanent neurologic deficit did not occur. Wound dehiscence was observed in a single patient (2%). Thirty-day and hospital mortality were not observed.

## Conclusions

Less invasive surgery on the aortic root, ascending aorta and aortic arch can be performed safely and reproducibly. Potential benefits include a minimized risk of wound dehiscence and reduced postoperative bleeding. The PUS does not compromise the quality of the operation.

